# Intra- and Interpolyelectrolyte Complexes of Polyampholytes

**DOI:** 10.3390/polym10101146

**Published:** 2018-10-14

**Authors:** Sarkyt E. Kudaibergenov, Nurxat Nuraje

**Affiliations:** 1 Laboratory of Functional Polymers, Institute of Polymer Materials and Technology, Almaty 050013, Kazakhstan; 2 Department of Chemical Engineering, Texas Tech University, Lubbock TX 79409−3121, Box 43121, USA

**Keywords:** polyampholyte, polyelectrolyte, interpolyelectrolyte complexes, intrapolyelectrolyte complexes, “isoelectric effect”

## Abstract

At present, a large amount of research from experimental and theoretical points of view has been done on interpolyelectrolyte complexes formed by electrostatic attractive forces and/or interpolymer complexes stabilized by hydrogen bonds. By contrast, relatively less attention has been given to polymer–polymer complex formation with synthetic polyampholytes (PA). In this review the complexation of polyampholytes with polyelectrolytes (PE) is considered from theoretical and application points of view. Formation of intra- and interpolyelectrolyte complexes of random, regular, block, dendritic polyampholytes are outlined. A separate subsection is devoted to amphoteric behavior of interpolyelectrolyte complexes. The realization of the so-called “isoelectric effect” for interpolyelectrolyte complexes of water-soluble polyampholytes, amphoteric hydrogels and cryogels with respect to surfactants, dye molecules, polyelectrolytes and proteins is demonstrated.

## 1. Introduction

Formation of polyelectrolyte-polyampholyte complexes (PPC) is crucial from fundamental and practical points of view [1,2,3,4,5]. IUPAC (International Union of Pure and Applied Chemistry) defines [6] that a polyampholyte (PA) is a special type of polyelectrolyte (PE) which possesses both cationic and anionic groups. Conditionally polyampholytes can be divided into 3 classes: “annealed”, “quenched” and “betainic” (or “zwitterionic”). “Annealed” polyampholytes consist of acid-base monomers that are ionized in dependence of pH, while “quenched” polyampholytes containing strongly charged cationic and anionic monomers retain their respective charges independently on pH. “Betainic” (or “zwitterionic”) polyampholytes are macromolecules containing identical numbers of acid-base (or fully charged anionic-cationic) species in the same monomer units [7,8,9,10]. The macromolecules existing via compensation of the cationic–anionic monomer pairs without counterions also belong to ”zwitterionic” polymers. In literature the terms “amphoteric” polyelectrolytes and “amphoteric” copolymers (or macromolecules) are also used [11]. Table 1 represents examples of various polyampholytes with different chemical structures. The microstructure of polyampholytes can be represented as random, alternating, graft, diblock, triblock, multiblock, dendritic, and stars.

Concerning the polyelectrolytes, we refer the readers to earlier publications [21,22,23,24,25,26,27,28,29], where the fundamental and applied aspects are described and to recent reviews [30,31], in which the theory of polyelectrolytes and a perspective on polyelectrolyte solutions are comprehensively outlined.

In the context of the present review, interaction of polyelectrolytes with polyampholytes leading to the formation of PPC is considered as a model reaction between biological objects, in particular between proteins and DNA [32], between sodium hyaulronate and proteins [33] on the one hand, and for protein purification and separation [34,35,36], enzyme immobilization [37], controlling delivery of DNA into the cell [38,39] on the other.

This review is mainly focused on the past, present and recent advances of intra- and interpolyelectrolyte complexes of polyampholytes. First, the theoretical achievements of polyelectrolyte–polyampholyte complexation are highlighted. The theory of polyampholytes is described briefly to introduce the readers to specific properties of amphoteric macromolecules in dependence of charge distribution. The ability of polyampholytes to form intrapolyelectrolyte complexes (Intra-PEC) at the isoelectric point (IEP) is considered. Then experimental findings on the interaction of polyelectrolytes with polyampholytes and formation of interpolyelectrolyte complexes (Inter-PEC) are reviewed. The realization of “the isoelectric effect” is discussed in the light of competition between the Intra- and Inter-PEC. Analogy between interpolyelectrolyte complexes derived from anionic and cationic polyelectrolyte pairs and polyampholytes is justified to expand our representation on amphoteric behavior of polyelectrolyte complexes.

## 2. Theory of Polyampholytes (PA) and Polyelectrolyte (PE)–Polyampholyte Complexation

### 2.1. Theory of Polyampholytes

Key problems of the protein-folding process definitely intensified the fast development of polyampholyte theory because the understanding of the protein-folding mechanism on the level of synthetic analogs can be of help to explain their structural–functional relationship [40,41,42,43,44,45,46,47,48,49,50,51,52,53,54,55,56,57,58,59,60,61,62,63,64,65,66,67,68,69]. Several attempts to simulate the folding of diblock polyampholyte chains were undertaken by authors [70,71,72,73,74]. The first theoretical work [41] considered the statistical polyampholytes containing *N* monomeric units with equal amounts of positive *f* and negative *g* charges and pointed out that fluctuation-induced attraction of opposite charges is the major factor determining their physicochemical behavior. This is called microelectrolyte and can be illustrated by the Debye–Huckel (DH) theory. The conformation change from collapsed state to extended one could be mathematically described by Equation (1):

(1) $$\begin{array}{cc} \gamma =\frac{e^{2}b^{2}}{kT}{\left(Ll\right)}^{-1/2}\; & \gg 1\;\mathrm{collapse}\\ & \ll 1\;\mathrm{open}\;\mathrm{collapse} \end{array}$$

In the above formula, *b l* and *L* represent the monomer size, the Kuhn length and the arc length, respectively, and *γ* represents dimensionless parameter. The rest of the physical parameters are the electron charge (*e*), the Boltzman constant (*k*), and the absolute temperature (*T*).

**Figure 1:**
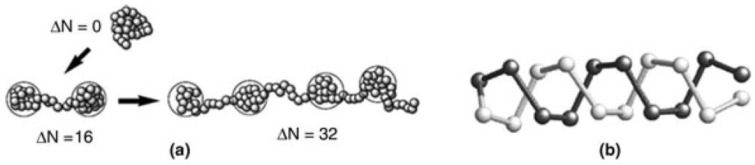
Cascade type of transition of polyampholyte chain in dependence of charge asymmetry (**a**) and double-stranded helical conformation of the ground state of a diblock polyampholyte obtained from Monte Carlo simulation (**b**). Dark and light shades indicate complementary charges (Reprinted from [72]).

Earlier research works [59,60] used DH theory to describe the behavior of polyampholytes which contain unevenly dispersed positive and negative charges. Collapsing phenomena among alternating polyampholytes is similar to precipitation of neutral macromolecules in poor solvents which experience a conformational transformation, “coil-globule”. The DH theory is not feasible for regular polyampholytes since the nearest-neighbor interactions of opposite charges act as dipoles and eliminate charge fluctuations [44]. Diblock polyampholytes containing the same length of flexible oppositely charged blocks collapse in three regimes [72]. In the first regime is the electrostatic repulsion of oppositely charged fragments stabilized by short-range repulsion between monomers. Globules can be represented as densely packed correlation blobs. These blobs are similar to correlation blobs of polyampholytes, which have a random distribution of positive and negative charges. The third regime is related to the formation of dipoles, which are associated into quadruples and further organized into big multiplets like ionomers [75]. Figure 1 demonstrated the evolution of polyampholytes (polymerization degree: *n* = 256).

A balanced polyampholyte with zero charge ΔN = 0 forms dense globule (where ΔN is the difference between the amount of positive fN^+^ and negative fN^−^ charges). Irrespective of positive or negative, at charge asymmetry (ΔN = 16) a globule is transformed into two halves as shown in Figure 1. Further increase in charge asymmetry (ΔN = 32) promotes the formation of “pearl” or bead strings connected by a “spring”. The simulation findings of variations in sequence and chain length of random, diblock and zwitterionic polyampholytes are consistent with experimental observations [65]. Comprehensive information on the theory of polyampholytes can be found in monograph [4].

All aforementioned theoretical conclusions successfully explain most experimental results.

(1) Polyampholytes demonstrate polycationic (if *f >> g*) or polyanionic (if *g >> f*) properties correspondingly. 

(2) At the isoelectric point (IEP) (if *f* ≈ *g*) the polyampholytes become quasi-electroneutral and have a small hydrodynamic radius. If soluble, they tend to aggregate; if insoluble, they precipitate.

(3) Addition of salts causes neutral polyampholytes swelling if polyampholytes are soluble, narrowing the phase separation region or even dissolving the precipitate if they are not soluble (“antipolyelectrolyte” effect).

However all aforementioned theories developed for polyampholytes consider mostly quenched polyampholytes; disregard the specific binding among counterions and polyion; consider mostly the diluted solutions and are applicable only at low concentration of salts; and do not take into account non-electrostatic forces (hydrogen bonds, hydrophobic interactions, Van der Waals forces). Nevertheless the aforementioned principles explain the solution behavior of most polyampholytes reasonably well.

### 2.2. Theory of Polyelectrolyte–Polyampholyte Complexation

The study of polyelectrolyte-polyampholyte complexation is promising for protein separation [76] and delivery of DNA into the cell [77]. The polyelectrolyte–protein or polyampholyte–DNA interactions are similar to the adsorption of polyampholytes on charged surfaces, because polyelectrolyte chains are able to polarize protein molecules and recognize them [3,78]. The Monte Carlo method [79] and molecular dynamics simulations [80,81] have been conducted to explain the polyampholyte–polyelectrolyte complexation as a function of charge distribution of a polyampholyte, nature of solvent and salt. The structure of polyelectrolyte-polyampholyte complexes strongly depends on the charge distribution of polyampholyte chain. The typical aggregate structures formed between random polyampholytes and polyelectrolytes (RPA-PE) on the one hand, and diblock polyampholyte and polyelectrolyte (DBPA-PE) on the other, show that DBPA forms micellar aggregate while RPA forms branch polymer-like aggregate. 

Molecular simulation study proved the contribution of polarization and indicated the stability order for the following complexes: block polyampholyte–polyelectrolyte > statistical polyampholyte–polyelectrolyte > alternating polyampholyte–polyelectrolyte.

**Figure 2:**
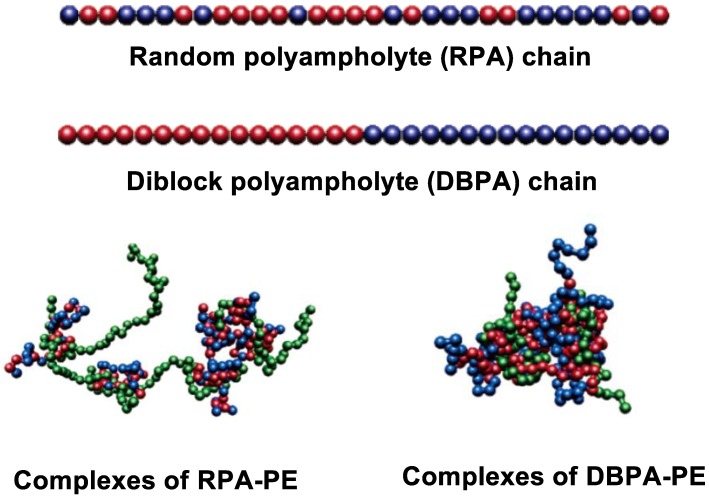
Snapshots of complexes of random polyampholyte (RPA) and diblock polyampholyte (DBPA) with polyelectrolyte (PE). Red bead represents the positively charged beads of polyampholyte; blue bead represents negatively charged ones; green bead represents polyelectrolyte chain. (Updated from [80]).

Computational works were devoted to (1) Monte Carlo simulation and analytical considerations on the adsorption of flexible polyelectrolyte chains onto charged Janus nanospheres (JNS) containing two oppositely charged hemispheres [82]; and (2) critical adsorption of periodic and random polyampholytes onto charged surfaces [83]. It is concluded that the PE chain adsorbed onto JNS differs from the adsorption of PE onto uniformly charged spheres. In the case of PA-surface adsorption, increasing PA block length enhances the adsorption onto charged planar surfaces (Figure 2). At low surface charge density *σ*, the oppositely charged blocks are internally bound to one another by electrostatic attractions and weakly bound to the surface. At high surface charge density, the positively charged blocks of PA strongly bind to the oppositely charged surface. In other words, the positive–negative interactions among the blocks within the PA are replaced by external binding of positive blocks with the negative surface and the negative PA blocks are liberated. After reaching the critical adsorption transition, *σ* *→* *σ*_c_, a rapid reduction in the binding energy occurs. It should be noted that under the same conditions and surface charge densities the binding energies for the periodic (block) PAs are significantly bigger than that of the random polyampholytes. Transformation of Inter-PEC to Intra-PEC is related to exchange of external contacts between PA blocks and PE chain to internal contacts between positively and negatively charged PA blocks. The simulation-based analysis of critical PA-surface adsorption conditions can successfully be applied for theoretical modeling of PE–PA, PE-protein, PE-RNA, and PE-DNA complexation. Electrostatic-driven binding of PE with Janus type amphoteric nano- or microgels may potentially be used as stimuli-responsive nano- and micro-actuators [84]. 

Monte Carlo simulations were used to study the pH effect on the behavior of isolated polyampholytes and PPC in dilute solutions [85]. Simulation for the polyelectrolyte-polyampholyte system show that the resulting complexes are stable at pH ≤ 6.5. A further increase in pH results in destruction of PPC due to repulsion between the polyampholyte and the negatively charged polyelectrolyte. This theoretical finding confirms the existence of the “isoelectric effect” that is realized at the IEP of polyampholytes and discussed in Section 5.

## 3. Intrapolyelectrolyte Complexes (Intra-PEC) of Polyampholytes

Formation of intrapolyelectrolyte complexes (Intra-PEC) within a single chain of polyampholytes is mostly pronounced for block polyampholytes [86]. A linear diblock copolymer poly(styrenesulfonate)-*b*-poly(2-vinylpyridine) (PSS-*b*-P2VP) is the first example of formation of Intra-PEC at the isoelectric point (IEP) of polyampholytes [87]. Depending on the degree of ionization of a P2VP block, the conformation of the PSS-*b*-P2VP is altered from segregated structure with a nucleus of P2VP blocks to a “frozen” coil and further to globular structure at the IEP due cooperative intraionic contacts between positively and negatively charged blocks. 

**Figure 3:**
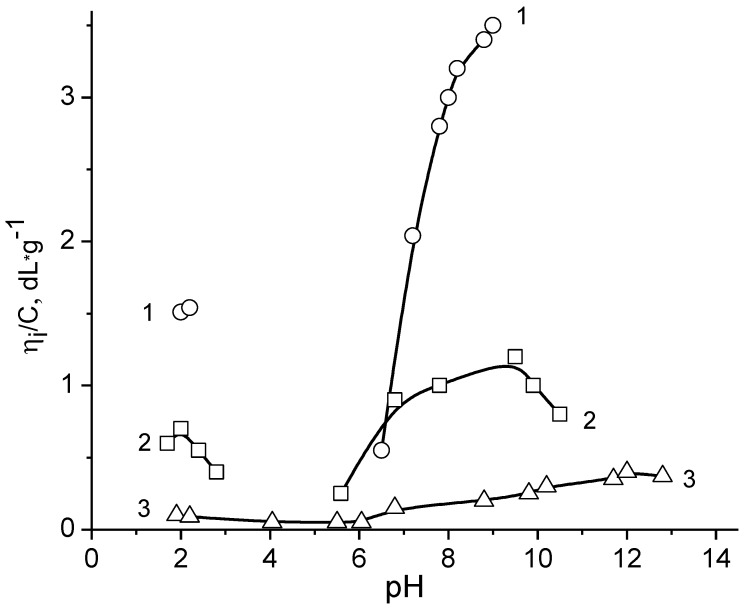
pH-dependent reduced viscosity of PMAA-*b*-P1M4VPCl in aqueous solution (1) and at ionic strength µ = 0.01 (2) and 0.3 mol·L^−1^ KCl (3). [PMAA-*b*-P1M4VPCl] = 0.045 g·dL^−1^. (Reprinted from [18]).

Similar behavior was observed for nearly equimolar BPA consisting of poly(methacrylic acid) (PMAA) and poly(1-methyl-4-vinylpyridinium chloride) (P1M4VPCl) [18,88]. As seen from Figure 3 in acidic and alkaline media PMAA-*b*-P1M4VPCl acts as polycation and polyanion, respectively; the reduced viscosity drops with the increase in the ionic strength (µ). 

**Scheme 1:**
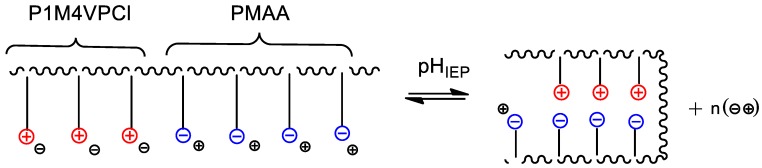
Formation of intrapolyelectrolyte complexes (Intra-PEC) at the isoelectric point (IEP) of equimolar PMAA-*b*-P1M4VPCl.

The insolubility of BPA in aqueous solution is observed at the IEP due to the formation of *globular* Intra-PEC because of cooperative interactions between the anionic and cationic blocks (Scheme 1).

By definition [89], a globule is the state of a macromolecule possessing a definite thermodynamically stable spatial structure in which the fluctuation of the density is rather small in comparison with the density itself, while the correlation radius of the density fluctuation is much smaller than the size of whole macromolecule. 

Increase in the ionic strength (µ) of the solution through addition of KCl diminishes the precipitation region and at higher values of µ = 0.3 mol·L^−1^ the BPA is entirely dissolved. Dissolution of BPA is due to screening of the electrostatic interactions among the oppositely charged blocks on the polymers by KCl, resulting in the change of globule to coil. By definition [89], coil is the state of macromolecule possessing an adequate spatial structure. In this case, the fluctuation of the density is in the order of density itself, while correlation radius is in the order of a macromolecular size. Thus, coil and globule differ from each other by fluctuation regime. From a statistical physics point of view, such a major (or big) difference between two states is called phases, and transformation between them is defined as a phase transition. According to this terminology, the transition of globular structure of BPA at the IEP to coil structure upon addition of low-molecular-weight salts can be qualified as a phase transition. 

**Scheme 2:**
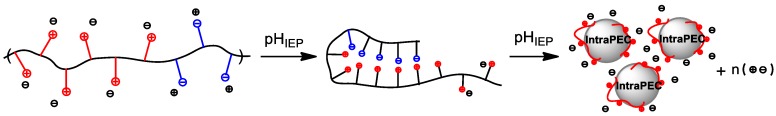
Schematic representation of Intra-PEC formed at the IEP of PMAA-*b*-P1M4VPCl with the excess of cationic block.

**Figure 4:**
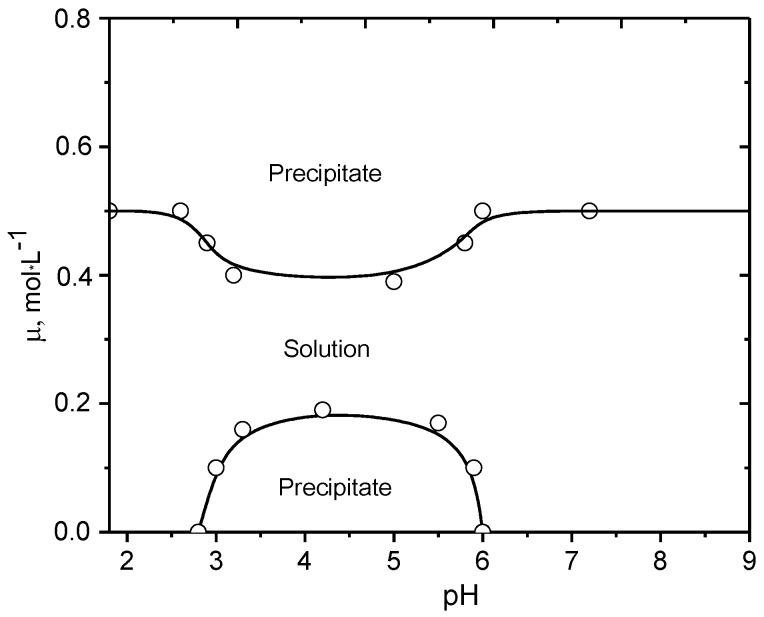
Phase diagram of PMAA-*b*-P1M4VPCl (45:55 mol %) in aqueous solution. [PMAA-*b*-P1M4VPCl] = 0.045 g·dL^−1^. (Reprinted from Ref. [18]).

A phase diagram of PMAA-*b*-P1M4VPCl at constant concentration of BPA (C = 0.045 g·dL^−1^) and various pH and ionic strength is shown in Figure 4. At the IEP BPA has a wide region of insolubility that is narrowed upon increase in the ionic strength of the solution adjusted by addition of KCl. The dissolution of BPA at µ = 0.2–0.4 mol·L^−1^ KCl is related to the destruction of the globules stabilized by intraionic salt “bridges” between the oppositely charged blocks. Precipitation of PMAA-*b*-P1M4VPCl at µ > 0.4 mol·L^−1^ can be explained by the “salting out” effect. The Intra-PEC of PMAA-*b*-P1M4VPCl composed of the excess of anionic or cationic blocks has the specific structure at the IEP [18,88]. Its aggregate is made of Intra-PEC surrounded by anionic (or cationic) blocks, which prevent it from precipitation and preserving the whole macromolecules in water-soluble state (Scheme 2). 

**Figure 5:**
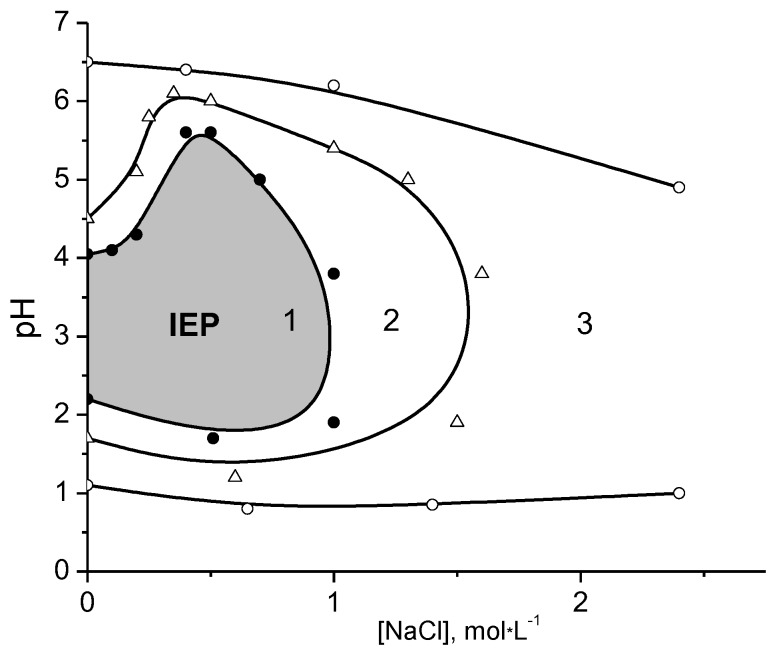
Phase diagram of chitosan carboxymethyl esters (CCM)-water-salt system as a function of polymer concentration. [CCM] = 0.05 wt % (1), 0.1 wt % (2) and 0.5 wt % (3).

Figure 5 presents the phase diagram of chitosan carboxymethyl esters (CCM)/water/salt systems with parameters including the polymer concentration, pH and ionic strength of the solution [90,91]. When the concentration of polyampholyte increases, an isoelectric region is broadened and separated into 2 phases. One is a solvent and the other is a swollen precipitated Intra-PEC. 

Formation of Intra-PEC at the IEP was compared for triblock and random polyampholytes based on *N*,*N*-dimethylaminoethylmethacrylate-methylmethacrylate-methacrylic acid (DMAEM-MMA-MAA) [92,93]. Light-scattering data of random and triblock copolymers DMAEM-MMA-MAA of 1:1:1 composition reveal that no aggregation is observed for random copolymers while large aggregates of the size 110–188 nm are formed for triblock copolymers at the IEP. The latter is due to the formation of globular Intra-PEC. The same phenomenon was observed for DMAEM-MAA containing hydrophobic spacers [94] and ABC triblock copolymers of poly(styrene)-*b*-poly(2- (or 4-)vinylpyridine)-*b*-poly(methacrylic acid) [95]. 

**Figure 6:**
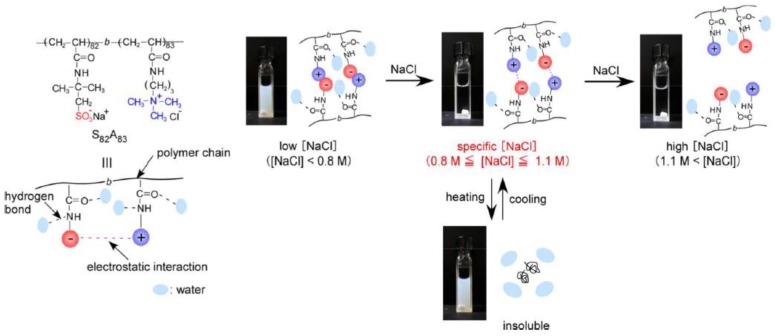
Thermo- and salt sensitivity of diblock “quenched” polyampholyte AMPSNa-APTAC. (Reprinted from [96]).

Diblock “quenched” polyampholytes composed of AMPSNa-APTAC synthesized by RAFT radical polymerization exhibit both thermo- and salt sensitive behavior [96]. At low salt concentration, the Intra-PEC is stabilized by ionic bonds (Figure 6). At 0.8 mol·L^−1^ ≤ [NaCl] ≤ 1.1 mol·L^−1^ the Intra-PEC is disrupted, becomes water-soluble and at 1.1 mol·L^−1^ ≤ [NaCl] transforms to unimer state with *R*_h_ = 6.0 nm. Upon heating, the saline solution of AMPSNa-APTAC shows the LCST (lower critical soluble temperature) due to enhancement of hydrophobic interactions of the polymer chains. 

Authors [97] studied the solution and volume-phase behavior, structural and stimuli-responsive properties of linear and crosslinked triblock polyampholytes PAA_109_-*b*-P2VP_819_-*b*-PAA_109_ and PAA_109_-*b*-QP2VP_819_-*b*-PAA_109_ (where PAA is poly(acrylic acid), P2VP is poly(2-vinylpyridine), QP2VP is quaternized P2VP) with the change in pH and ionic strength of the solution together with computer simulation of the systems. At pH > 5.0 the system PAA_109_-*b*-QP2VP_819_-*b*-PAA_109_ forms a viscous opaque liquid, while at pH < 3.0 it forms a stiff gel. These results are interpreted in terms of a charge-driven association between oppositely charged blocks and the formation of neutral hydrophobic complexes. 

The rheology, rotational dynamic light scattering and small angle neutron scattering were used to evaluate the influence of the pH on gel and solution behavior of the triblock terpolymer: poly(methyl methacrylate)-*b*-poly(2-(diethylamino)ethyl methacrylate-*co*-methacrylic acid)-*b*-poly(methyl methacrylate) (PMAA-*b*-P(DEA-MAA)-*b*-PMMA) [98,99]. Existence of telechelic polyampholytes in several regimes was identified. Phase separation, or precipitation, of PMAA-*b*-P(DEA-MAA)-*b*-PMMA was observed at the IEP (in the range of pH 7.6–8.8). At pH 6.0–7.4 (just below the IEP regime) a sol–gel transition was fixed. In acidic or basic regions, amphoteric macromolecules are in stretched conformation due to predominance of positive or negative charges in the polymer chain. 

Recent remarkable review [100] is focused on the comparative analysis of stimuli-responsive hydrogels made of block co-polyampholytes and block copolymers or homopolyelectrolytes. The main attention is paid to physically crosslinked hydrogels based on the intermolecular association of hydrophilic triblock copolymers end-capped by polyion sequences. Another gelling system is a mixture of triblock copolymer with ionic end blocks and an oppositely charged homopolymer. The study of the solution, rheological and structural properties of co-assembled hydrogels offers important guidance for their potential biomedical application. 

The globular structure of macromolecules [89] can be considered as dense three-dimensional nucleation, consisting of a “core” from which the monomers “force out of” the solvent and surrounded by a hydrophilic “edge”. Such globular structure can be realized at the IEP of water-soluble polyampholytes and lead to formation of high ordered structures [101]. In the authors’ opinion, the appearance of sharp lines in a Raman spectrum, especially in the low-frequency region, indicates the presence of a high-ordered structure like crystalline domains in aqueous media. However, additional XRD (X-ray diffraction), SAXS (small angle X-ray scattering), WAXS (wide angle X-ray scattering), SANS (small angle neutron scattering) measurements are needed to confirm such hypothesis. 

## 4. Interpolyelectrolyte Complexes of Polyampholytes

**Scheme 3:**
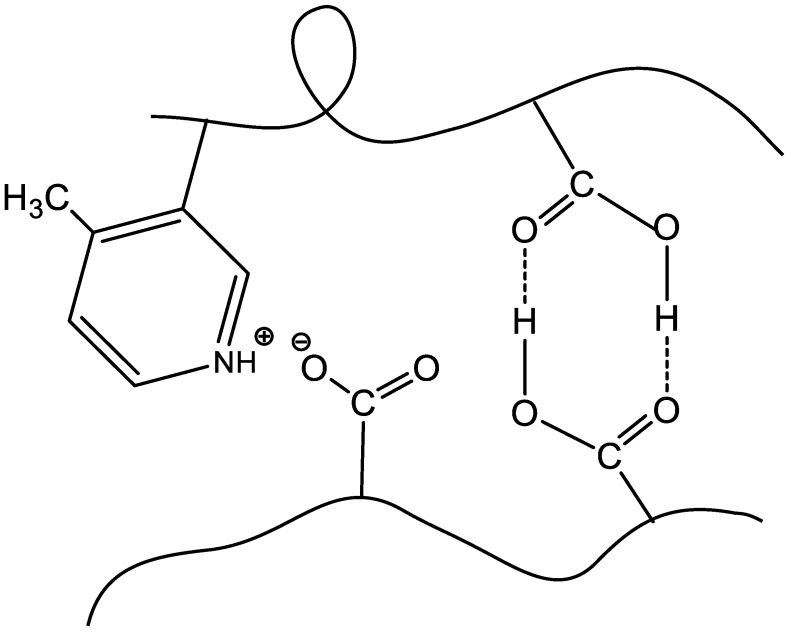
Schematic representation of Inter-PEC between 2-methyl-5-vinylpyridine-*co*-acrylic acid (2M5VPy-*co*-AA) and poly(acrylic acid) (PAA).

The pioneering works of H.A. Bixler and A.S. Michaelis [102], V.A. Kabanov [103], B. Philipp [104], E. Tsuchida [105], and E.A. Bekturov [106] made substantial contributions to the fast development of interpolyelectrolyte (or interpolymer) complexes. Various aspects of polyelectrolyte–polyelectrolyte complexes have been reviewed recently [107,108,109,110,111]. Interpolyelectrolyte complexes of polyampholytes [112] are a subject less considered in comparison with hydrogen-bonded interpolymer complexes [113,114]. The complexation between 2-methyl-5-vinylpyridine-acrylic acid (2M5VPy-*co*-AA) and poly(acrylic acid) (PAA) was first studied by V.A. Kabanov et al. [115]. It was established that the common cooperative system consisting of the 2M5VPy-*co*-AA and PAA via ionic and hydrogen bonds is responsible for PPC formation (Scheme 3). 

**Figure 7:**
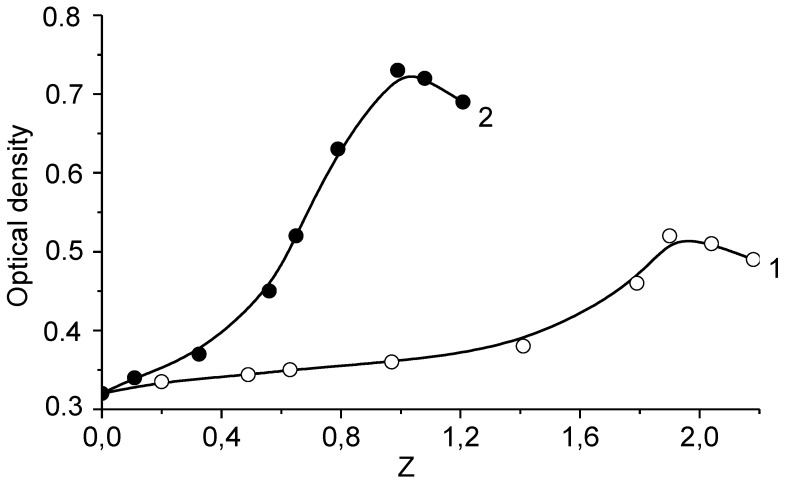
Turbidimetric titration curves for ionene bromide solution by chitosan sulfate (CS) at pH = 2.5 (curve 1) and 11.5 (curve 2). [2,5-ionene] = 5·10^−4^ mol·L^−1^. *Z* = [CS]/[2,5-ionene].

The interaction between polyampholyte and 2,5-ionene bromide by turbidimetry was studied by [116] (Figure 7). 

It was found that in the alkaline region Inter-PEC while in acidic region Intra-PEC is formed.

The competition between the Intra-PEC and Inter-PEC is clearly seen when ^13^C nuclear magnetic resonance (NMR) spectra of *N*-methyldiallylamine-maleic acid (MDAA-MA) and mixture of MDAA-MA and poly(*N*,*N*-dimethyl-*N*,*N*-diallylammonium chloride) (PDMDAAC) are compared [117,118,119,120,121]. The signals of carboxylate anions of MDAA-MA participating in the formation of Intra-PEC appear at 180.5 and 180.8 ppm. The formation of Inter-PEC between carboxylate anions of MDAA-MA and quaternary nitrogen atoms of PDMDAAC shifts the same peaks to 181.3 and 181.8 ppm. At the IEP of *N*,*N*-dimethyldiallylammonium-maleic acid (DMDAA-MA) (pH_IEP_ = 4.1), the position of the carboxylate anions peak of DMDAA-MA at 182.4 ppm does not change during the addition of PDMDAAC, e.g., the strength of Intra-PEC is higher than that of the Inter-PEC. Formation of the Inter-PEC between cellulose-based poly(zwitterion) and PDMDAAC is mentioned in [122].

**Scheme 4:**
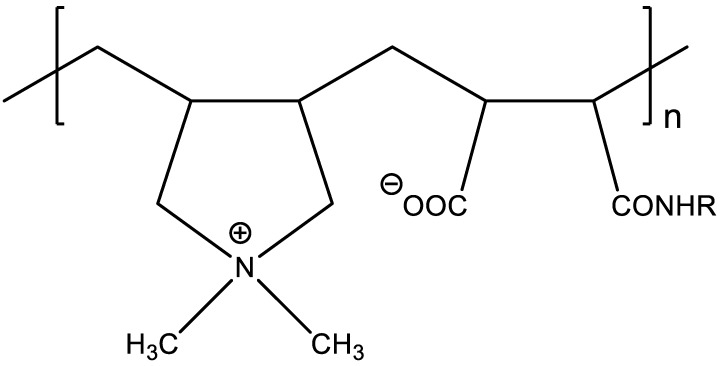
The repeating units of APA (Alkyl (or aryl) polyampholytes) series, where *R* = H (APA-1); C_4_H_9_ (APA-2); C_6_H_5_ (APA-3); C_6_H_5_(CH_2_)_3_CH_3_ (APA-4) (Reprinted from [123]).

A copolymer of *N*,*N*-dimethyldiallylammonium and alkyl (or aryl) derivatives of maleamic acids (abbreviated as APA series in dependence of the length of the hydrophobic moieties of maleamic acid) were involved into the complexation with PAA and poly(styrene sodium sulfonate) (NaPSS) [123] (Scheme 4) 

The PPCs with participation of PAA (or NaPSS) and polyampholytes of the APA series are very compact and represent core–shell particles that are preserved in aqueous solution due to the presence of carboxylic and amide groups of APA. Table 2 summarizes the compositions of PPC formed between APA series and PAA or NaPSS.

Interaction of amphoteric dendrimers and proteins with linear and crosslinked anionic and cationic polyelectrolytes was investigated by Kabanov et al. [124,125]. In acidic and alkaline media up to pH ~ pH_IEP_, dendritic polyampholytes can form Inter-PEC with flexible linear polyanions and polycations. The polyampholyte dendrimers are also efficiently sorbed by a polyanionic (below the IEP) and polycationic hydrogels (above the IEP). The binding degree of polyampholyte dendrimers with oppositely charged linear or crosslinked polyelectrolytes is ascribed to the competition between intra-dendrimeric zwitterions and interionic salt bonds of functional groups of dendrimers and polyelectrolytes.

**Figure 8:**
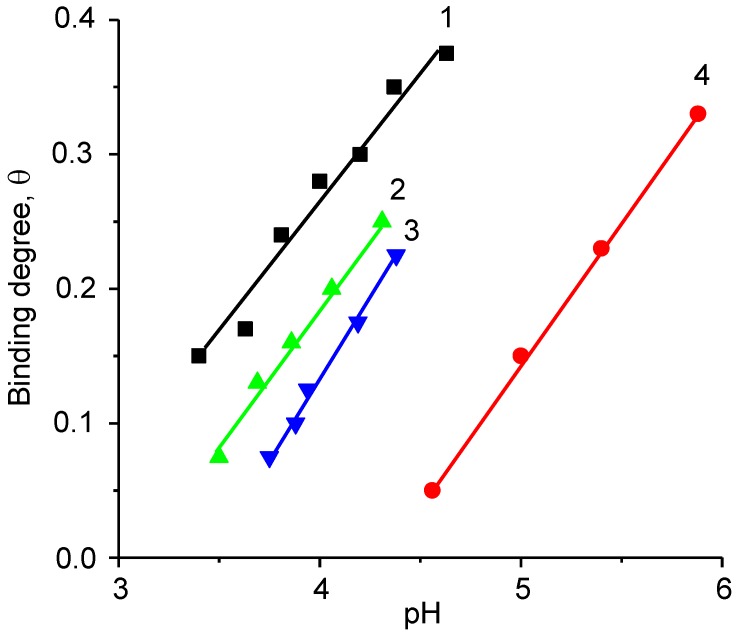
pH profile of binding degree θ for systems consisting of PMAA-*b*-P1M4VPCl/PAA (1), PMAA-*b*-P1M4VPCl/PDMDAAC (2), (PMAA-*b*-P1M4VPCl/PDMDAAC)/PAA (3). Curve 4 corresponds to intramolecular complexation within block polyampholyte PMAA-*b*-P1M4VPCl. (Reprinted from Ref. [88]).

The BPA binds the anionic and cationic polyelectrolytes more cooperatively in comparison with statistical and alternating polyampholytes [17,18,88]. Complexation between BPA and anionic (or cationic) polyelectrolyte is higher than complexation between the acidic and basic blocks of BPA. It should be mentioned that BPA binds the cationic (or anionic) initially and later the anionic (or cationic) polyelectrolytes (Figure 8). 

**Figure 9:**
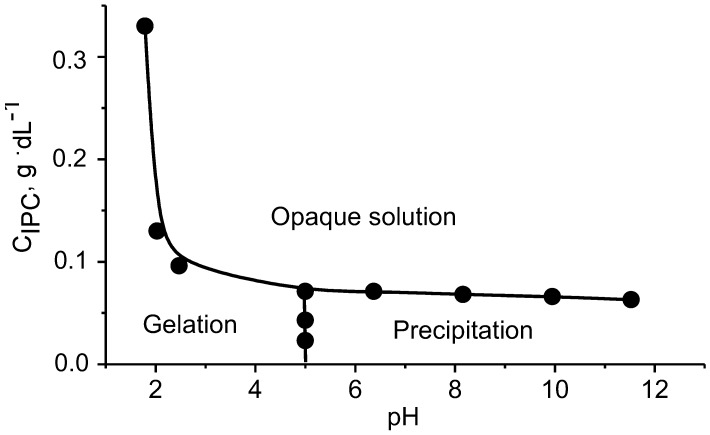
Phase transitions of Inter-PEC consisting of block polyampholyte PMAA-*b*-P1M4VPCl and poly(vinylbenzyltrimethylammonium chloride) (PVBTMACl) in water with opaque solution, gelation, and precipitation regions. (Reprinted from [18]).

However, the cooperative character of this process is much lower than the cooperativity of block polyampholyte–polyelectrolyte pairs. Such Inter-PECs are insoluble and tend to precipitate. Figure 9 shows the phase diagram of the system consisting of PMAA-*b*-P1M4VPCl and PVBTMACl in dependence of PMAA-*b*-P1M4VPCl concentration and pH of the solution. 

**Scheme 5:**
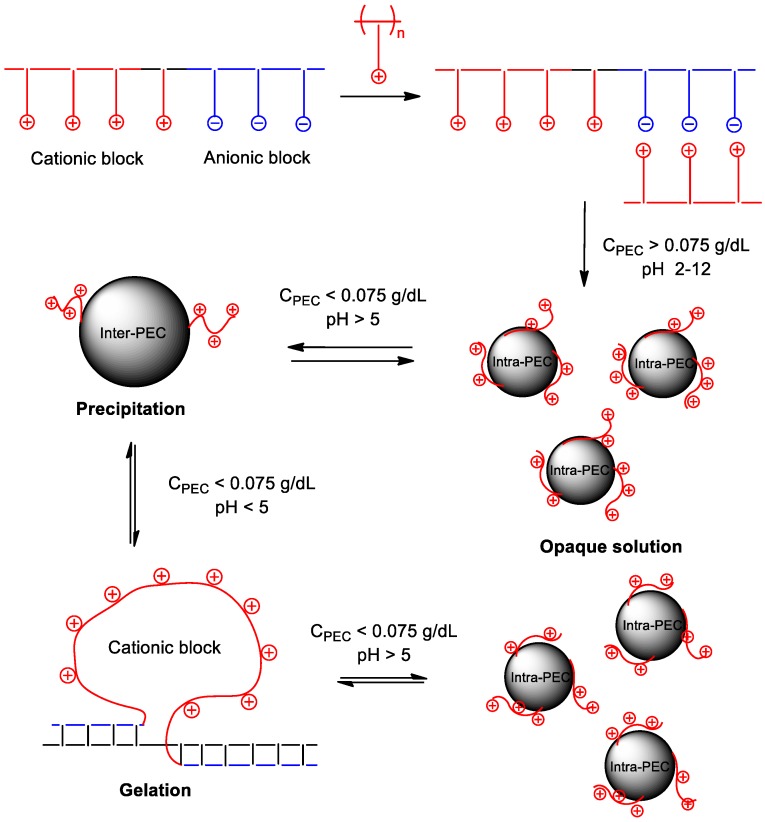
Schematic representation of phase transitions of system PMAA-*b*-P1M4VPCl/PVBTMACl in dependence of BPA (blockpolyampholyte) concentration and pH of the solution.

The Inter-PEC in the form of opaque solution is formed at C_PEC_ > 0.075 g·dL^−1^ and pH = 2–12. The precipitation starts at pH ≥ 5.0 and C_PEC_ <0.075 g·dL^−1^. The gelation occurs preferentially at pH < 5.0 and C_PEC_ > 0.075 g·dL^−1^. Thus there are three phases in this system which are solution, gel and precipitate. They can be transformed into each other with changes in either PEC concentration or pH. Higher C_PEC_ leads to precipitation of inter-PEC particles. At the relatively low C_PEC_ they are more uniformly distributed to form an opaque solution. Both the phase transition between precipitate–microgel and between opaque solution–microgel at low pH are subject to the swelling of Inter-PEC particles and decrease in the interaction among the ionized carboxylic groups of acidic blocks in Inter-PEC formation. The gel formation process occurs following the release of PMAA “loops” which form interchain networks with H-bonding. On the basis of the above results, the transitions between precipitate–opaque solution, precipitate-microgel and opaque solution–microgel can be illustrated as shown in Scheme 5:

A phase diagram for triblock and random polyampholyte AA-DMAEM-MMA (0.9:1:1) and poly(vinyl alcohol) (PVA) was investigated along with polymer concentration, pH, and salt concentration [126,127]. Phase separation occurred at 10% polymer concentrations. The phase diagrams of PVA with the block polyampholytes exhibit whole miscibility at acidic and alkaline pH regardless of salt concentration. Each region of the phase diagram was interpreted in terms of the formation of cooperative hydrogen bonds between the hydroxyl groups of PVA and carboxyl groups of the undissociated methacrylic acid residue of polyampholytes. PVA and the random polyampholytes generally display one-phase behavior in the pH-(KCl) space. The phase behavior of the triblock polyampholytes with PVA is useful to apply for biological molecule separation including proteins, and organic molecule contaminants, such as halogenated aromatic hydrocarbons. 

**Scheme 6:**
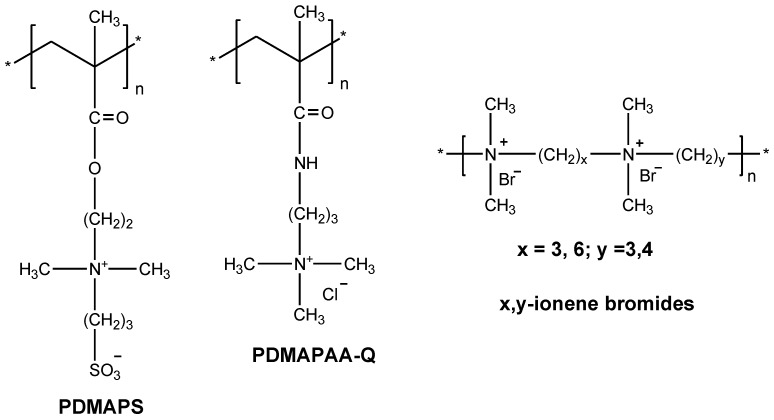
Chemical structures of polymers used for complexation reactions (Reprinted from [128]).

Authors [128,129] studied the complexation of polycarbobetaines with NaPSS, DNA and poly(zwitterion)—poly[3-dimethyl(methacryloyloxyethyl ammonium propane sulfonate)] (PDMAPS) with polymeric anion: poly(2-acrylamido-2-methyl propane sulfonic acid) (PAMPS) or polymeric cations: poly(3-acrylamidopropyltrimethyl ammonium chloride) (PDMAPAA-Q) and *x,y*-ionene bromides (*x* = 3, 6; *y* = 3,4) (Scheme 6). 

**Scheme 7:**
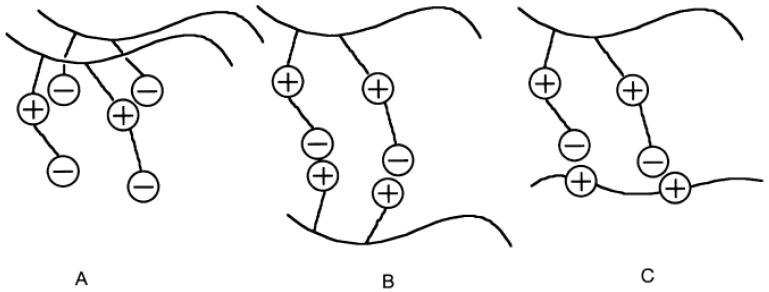
Scheme of formation of Inter-PEC between PDMAPS and PAMPS (**A**), PDMAPAA-Q (**B**) and x,y-ionene bromides (**C**). (Reprinted from [128]).

The phase behavior of PDMAPS is totally altered with the addition of these polyelectrolytes, and the resulting UCST (upper critical soluble temperature) values are found to be different. Furthermore, the UCST decreased significantly with addition of PAMPS into the PDMAPS solution and vanished at high PAMPS concentration. This distinct variation in the UCST indicates the strong interaction between two polyelectrolytes which is correlated with the geometrical structure of polyelectrolyte complexes (Scheme 7A–C). 

In the case of PDMAPS–PAMPS, an ammonium cation in the middle interacts with sulfonate anion of PAMPS. Such Inter-PEC is solubilized in water by free anionic groups of PDMAPS (Scheme 7A). For solubilization of Inter-PEC consisting of PDMAPAA-Q and PDMAPS, ammonium cation of PDMAPS is responsible because the sulfonates located at the end of side chain are occupied by quaternary ammonium groups of PDMAPAA-Q (Scheme 7B). Also, PDMAPS can interact with ionene through the sulfonates located at the end of side chain (Scheme 7C). Thus, complexation of PDMAPS with anionic polyelectrolyte drastically increases the viscosity via forming a network and reduces the UCST with increase in the molar ratio of PAMPS/PDMAPS, which is ascribed to presence of free sulfonates located at the end of side-chain. However, the complexation of PDMAPS-polycation first minimizes the UCST, which is also found in the complexation of PDMAPS-PAMPS, then the UCST value increases with a molar ratio of polycation/PDMAPS without forming the network due to the presence of the free ammonium cation of PDMAPS in the middle of the side chain.

**Figure 10:**
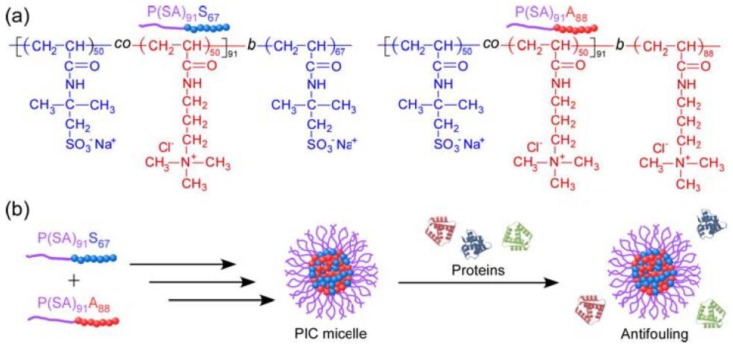
Chemical structure of random copolymers of AMPSNa-APTAC containing the anionic (AMPSNa) and cationic (APTAC) blocks (**a**) and schematic representation of Inter-PEC micelle formation together with protein antifouling properties (**b**). (Reprinted from Ref. [132]).

The formation of Inter-PEC micelles and vesicles was described by [130,131,132]. Micelles assembled from random copolymers of AMPSNa-*co*-APTAC [132] end-capped by anionic (AMPSNa) or cationic (APTAC) blocks that show protein antifouling properties (Figure 10). 

**Figure 11:**
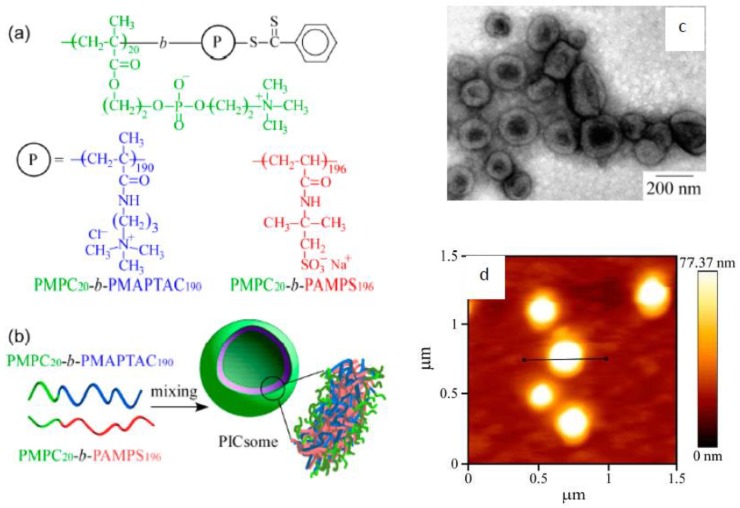
Chemical structures of PMPC-APTAC and PMPC-AMPSNa diblock copolymers (**a**), conceptual illustration of stoichiometric Inter-PEC vesicle formation (**b**), transmission electron microsope (TEM) (**c**) and atomic force microscope (AFM) (**d**) images of vesicles. (Reprinted from [131]).

Inter-PEC vesicles were assembled by mixing aqueous solutions of diblock copolymers consisting of a hydrophilic poly(2-(methcaryloyloxy)ethylphosphorylcholine (PMPC) block and either a cationic (APTAC) or anionic (AMPSNa) blocks (Figure 11). 

Both Inter-PEC micelles and vesicles may be appropriate carriers for bioactive compounds and used as drug delivery systems.

**Figure 12:**
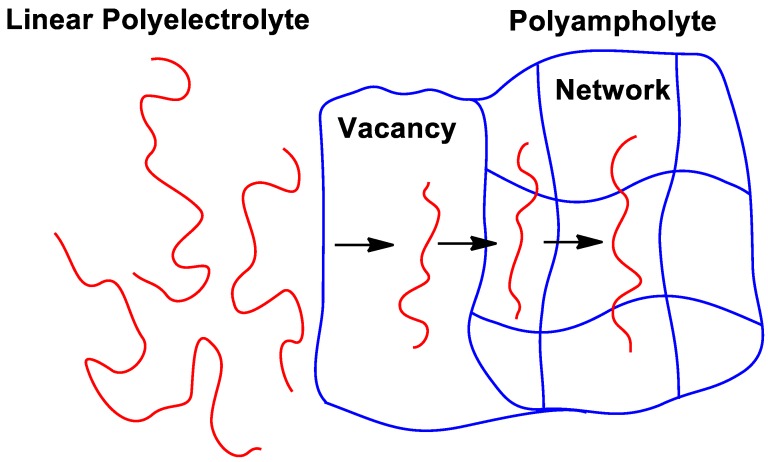
Penetration of macromolecules into hydrogel matrix via “ion-hopping transportation” mechanism.

Complexation of polyampholyte gels and linear polyelectrolytes is a less considered subject [107]. Mechanism of sorption of polyelectrolytes by amphoteric gel is similar to the penetration of linear polyelectrolytes within the oppositely charged polyelectrolyte networks, as shown schematically in Figure 12.

Penetration of macromolecules into hydrogel matrix proceeds via “race-relay ion transport” (or “ion-hopping transportation”) mechanism resulting in gel contraction. The driving force of this process is electrostatic binding of anionic or cationic polyelectrolytes with positive or negative charges of hydrogels, e.g., constant migration of charged macromolecules deeply into the gel volume by exchanging one fragment of the network to another vacant place. Each transfer taking place at the gel–solution interface is directed towards the hydrogel phase and leads to the formation of a vacancy within network that in turn is accessible to other macromolecules for transfer in the same direction.

**Figure 13:**
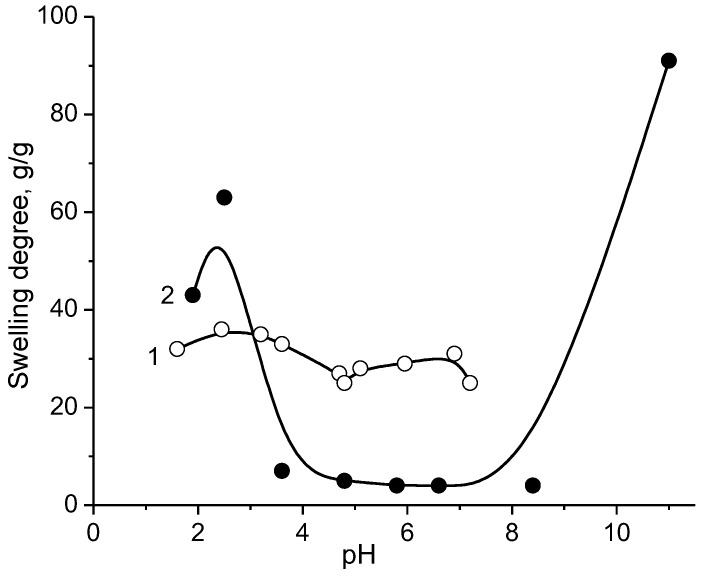
pH dependent swelling of amphoteric gel MA-DMDAAC-DAA (1) and complexes of amphoteric gel with NaPSS (2). (Updated from [133]).

The swelling–deswelling behavior of amphoteric gel which was made of maleic acid (MA), *N*,*N*’-dimehyldiallylammonium chloride (DMDAAC) and diallylamine (DAA) was studied in the presence of NaPSS [133]. The swelling degree of the amphoteric gel–NaPSS is minimally in the pH range between 4 and 8 (Figure 13) and increases significantly in the strong acidic and alkaline regions. As distinct from amphoteric gel MA-DMDAAC-DAA, which shrinks at the IEP (pH ≈ 4.6), the complex of MA-DMDAAC-DAA with NaPSS collapses over a wide range of pH because the NaPSS entrapped in the gel network acts as additional physical crosslinker. The complexation of polyampholyte gel with linear polyelectrolyte considerably broadens the IEP of PPC in comparison with the IEP of amphoteric gel itself. 

Layer-by-layer (LbL) assembling of rigid polyampholyte—sulfonated cardo poly(arylen ether sulfone) (SPES)—with anionic NaPSS and cationic PDMDAAC was shown by [134]. The formation of LbL between SPES and NaPSS was not observed in the pH range of 1~12. In contrast, multilayers were easily fabricated between SPES and PDMDAAC in the same pH range. The reason is that SPES contain two negative charges and one positive charge with every repeat unit exhibiting preferentially polyanionic behavior. The remarkable behavior of polyampholytes to change the net charge from positive to negative through the IEP is a powerful tool to assemble the LbL coatings or films with both cationic and anionic polyelectrolytes. Such an approach was realized by authors [135] to obtain pH-sensitive LbL films with an amphoteric copolymer, i.e., poly(diallylamine-co-maleic acid) (PDAMA). LbL films between the NaPSS and PDAMA were formed at pH 3.0, while LbL films containing PDAMA and cationic poly(*N*-ethyl-4-vinylpyridine) (PEVP) were observed at pH 8.0. Using this methodology, the same authors [136] were able to use PDAMA as a component to construct free-standing LbL films in combination with the NaPSS or the PDMDAAC. The resulting films were coated onto a quartz slide at a given pH value of 4.0 and 8.0. Furthermore, the precursor, or so called sacrificial layer, was covered with LbL films of poly(allylamine hydrochloride) (PAH) and NaPSS. After pH dependent dissolution of the PDAMA-PDMDAAC or PDAMA-NaPSS precursor layer, the resulting free-standing PAH-PSS films are colloidally stable in water for a few months. If the films are thick enough, they can be stable in air.

## 5. Realization of the ‘Isoelectric Effect’ at the Isoelectric Point (IEP) of Polyampholytes

At the IEP the compact globule structure is formed via squeezing out the solvent due to strong electrostatic attraction forces between oppositely charged groups. The isoelectric effect taking place at the IEP was first reported by authors [137,138,139] and afterwards confirmed by several researchers [140,141,142]. Moreover, it can be stated that any molecules associated with polyampholytes can potentially be discharged at the IEP via controlling their inter- and intramolecular interactions. Furthermore, if intrachain interaction of acidic and basic groups within a single macromolecule prevails the above releasing phenomena can be realized via overcoming interchain interaction between substance and polyampholytes. The realization of the “isoelectric effect” for a series of water-soluble polyampholytes was demonstrated for metal ions [143], dye molecules [144], polyelectrolytes and proteins [145]. 

**Figure 14:**
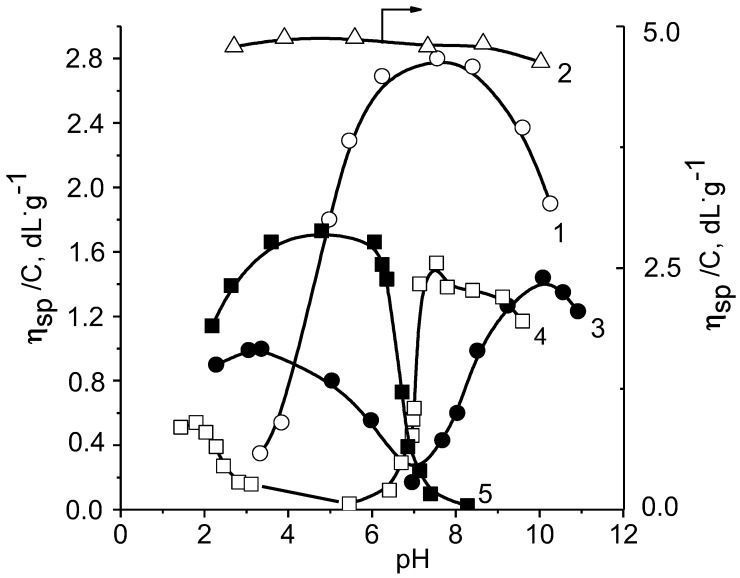
Effect of pH on the reduced viscosity of PAA (curve 1), PDMDAAC (curve 2), DMVEP-*co*-AA (curve 3), and Inter-PEC composed of DMVEP-*co*-AA/PAA (curve 4) and DMVEP-*co*-AA/PDMDAAC (curve 5) in water.

The existence of the “isoelectric effect” was clearly shown for systems consisting of polyampholyte based on 2,5-dimethyl-4-vinylethynylpiperidinol-4 and acrylic acid (DMVEP-*co*-AA) and anionic or cationic polyelectrolytes [145]. The composition of Inter-PEC derived from the potentiometric, conductimetric and turbidimetric curves is identified to be at the ratio of [polyampholyte] to [polyelectrolyte] = 3:1. Figure 14 shows the correlation of the reduced viscosity with pH for the individual components and Inter-PEC.

**Figure 15:**
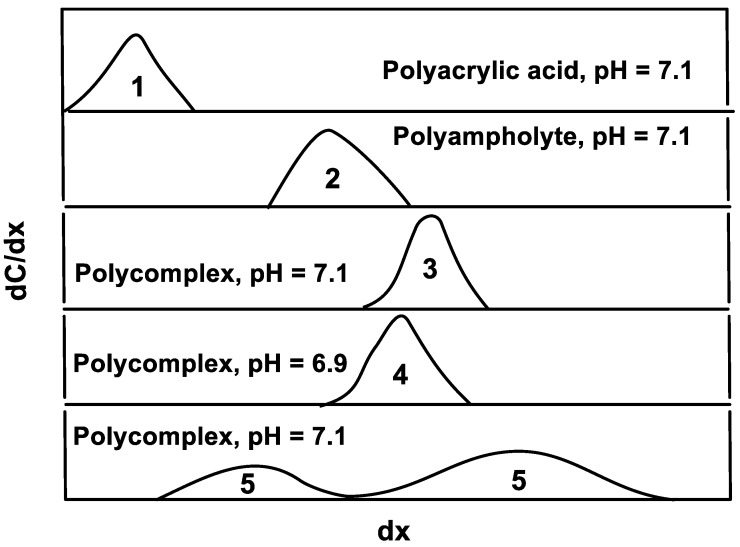
Sedimentograms of PAA (curve 1), DMVEP-*co*-AA (curve 2) and polyelectrolyte complexes composed of [DMVEP-*co*-AA]/[PAA] = 3:1 (curves 3-5) at pH = 6.1 (curve 3), 6.9 (curve 4), and 7.1 (curves 1, 2, 5).

Phase separation for the following two systems occurs at the different pH ranges. For the system composed of DMVEP-*co*-AA and PAA it occurs at 3.0 < pH < 5.5 and it occurs at pH > 8.2 for the mixture of DMVEP-*co*-AA and PDMDAAC. Near the IEP of DMVEP-*co*-AA (pH_iep_ = 7.0) the reduced viscosity of associates increases sharply. This is due to destruction of polycomplex particles into individual components. A cooperative destruction of polycomplex particles near the IEP is confirmed by an independent sedimentation experiment. Figure 15 shows the sedimentation diagrams of DMVEP-*co*-AA, PAA and polycomplexes at various pH. 

**Scheme 8:**
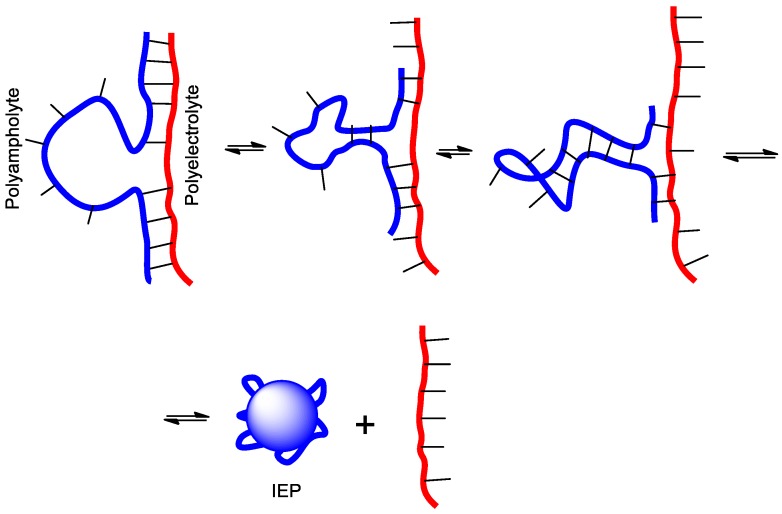
Proposed mechanism for destruction of Inter-PEC near the IEP of polyampholyte.

The sedimentograms of the polyampholyte–polyelectrolyte complex demonstrate a single peak with sedimentation coefficient *S* = 4.75 and 4.18 at pH = 6.1 and 6.9, respectively. However at pH = 7.1 two peaks with *S* = 1.9 (“slow peak”) and 5.6 (“fast peak”) show up. The sedimentation coefficients for individual PAA and DMVEP-*co*-AA at pH = 7.1 are 1.06 and 3.53, respectively. Thus the presence of “slow” and “fast” sedimentation peaks at narrow interval of pH (∆pH = 0.2) is ascribed to the destruction of Inter-PEC into the singular macromolecules. Inter-PEC consisting of polyampholyte and polyelectrolyte is expressed as double strand sequences of pairs formed via ionic and hydrogen bonds [108]. At the near IEP some acidic and basic groups of polyampholytes displayed on loops can interact with each other and form Intra-PEC. Scheme 8 represents the speculative mechanism of destruction of polyampholyte–polyelectrolyte associates near the IEP. 

The dependence of Inter-PEC on pH provides further evidence for the validity of the proposed mechanism [141]. The yield of Inter-PEC composed of polyampholyte-poly(styrenesulfonate) (PA-NaPSS) and polyampholyte-poly(N,N-dimethyldiallylammonium chloride) (PA-PDMDAAC) is close to zero at the IEP of polyampholyte.

**Scheme 9:**
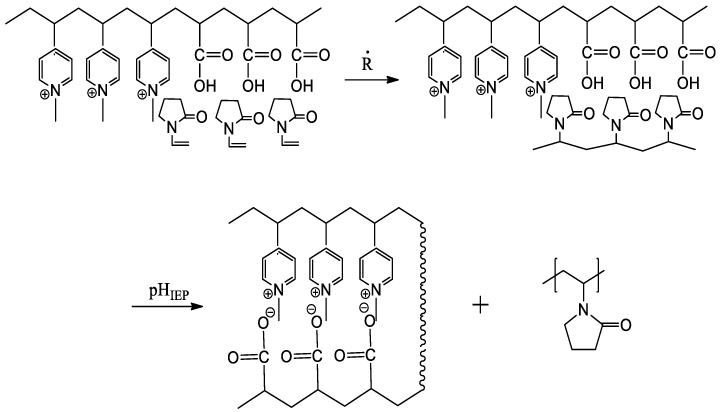
Realization of the “isoelectric effect” in synthesis of poly(*N*-vinylpyrrolidone) on blockpolyampholyte matrix.

The “isoelectric effect” can also smartly be used for matrix polyreactions [146]. Scheme 9 explains the synthesis reaction of poly(*N*-vinylpyrrolidone) on block polyampholyte matrix. Application of block polyampholyte as polymerization matrix has the following advantages in comparison with polyacids and polybases alone: (1) each block (anionic or cationic) can serve as polymerization matrix; (2) the product of matrix polymerization is water-soluble and preserved from the precipitation due to free (non-loaded) acidic or basic blocks; (3) the “daughter” chain can easily be separated from the matrix as a result of competition between inter- and intramacromolecular complexation; (4) molecular weight of products can be to some degree controlled by the length of polymer matrix; (5) block polyampholyte can be used for matrix polyreactions several times. 

The method of protein separation by water-soluble polyampholytes is selective complexation of polyampholyte with one of the proteins, which has predominating positive or negative charge [93,147,148,149,150]. Upon addition of polyampholyte to a mixture of proteins, some proteins form a complex with the polyampholyte, as the others stay in the supernatant phase [151]. The precipitated protein–polyampholyte complex may be isolated from the system and then redissolved at a different pH or else can be destroyed at the IEP, where the full precipitation of polyampholyte itself takes place [152].

**Scheme 10:**
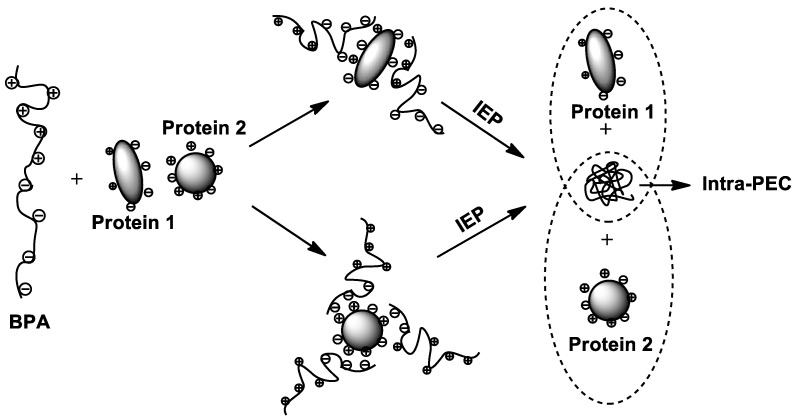
Separation of protein mixture at the IEP of BPA.

Unique separation of protein mixture was performed at the IEP of block polyampholytes [153]. In this case one of the blocks of polyampholyte interacts with one charge of protein, which is opposite to its charge, while the main chain (or other block)—with another protein charge as illustrated in Scheme 10.

In both cases protein release process occurs at the IEP of polyampholyte due to the formation of intraionic polyelectrolyte complexes (Intra-PEC).

**Figure 16:**
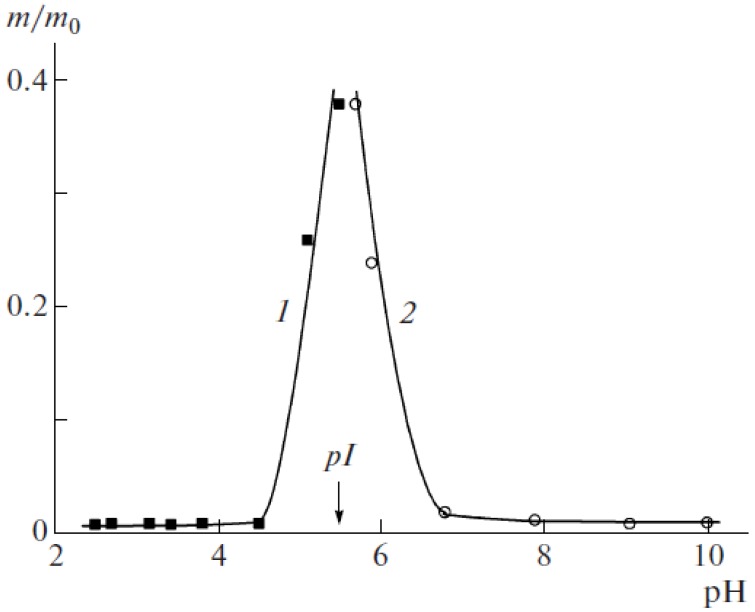
pH dependence of relative weight *m/m_0_* for polyampholyte dendrimer-PAMPSNa (1) and polyampholyte dendrimer-PDMDAAC (2) complexes. *pI* denotes the isoelectric point. (Reprinted from [154]).

It is interesting to note that the “isoelectric effect” was observed for complexes of polyampholyte dendrimers with highly swelling sodium poly(2-acrylamido-2-methylpropanesufonate) (PAMPSNa) and PDMDAAC used as polyelectrolyte networks [154]. Polyampholyte dendrimers represent the carboxylated poly(propylenimine) dendrimers (PPID) of five generations abbreviated as PPID-(COOH)*_x_*, where *x* = 4, 8, 16, 32 and 64. As seen from Figure 16 at the IEP of polyampholyte dendrimer the relative weight of Inter-PEC samples *m/m*_0_ (where *m* is the equilibrium weight of the product of completed sorption at a given pH of the equilibrium solution, *m*_0_ is the weight of initial equilibrium swollen gel in water) increases significantly due to destruction of Inter-PEC and release of polyampholyte dendrimer to outer solution from the polyelectrolyte network. If such a sample is washed with distilled water, its relative weight becomes close to unity.

**Scheme 11:**
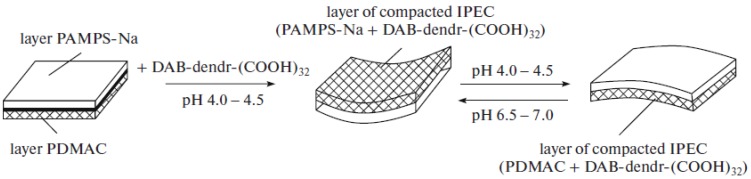
pH dependent transfer of polyampholyte dendrimer from anionic gel to cationic one through the IEP. (Reprinted from [154]).

Thus at the IEP of polyampholyte dendrimer the detachment of amphoteric macromolecules from the gel matrix of polyelectrolytes takes place. This statement is very important because the “isoelectric effect” can be realized not only for linear polyampholyte–polyelectrolyte complexes, but also for water-soluble dendrimeric polyampholyte-polyelectrolyte gels. The mechanism of release of polyelectrolytes or polyampholytes can be explained by competition between Intra-PEC and Inter-PEC formation. The data of Figure 16 suggest that the polyampholyte dendrimer can pass from the Inter-PEC containing the anionic gel to a new Inter-PEC formed by the cationic gel, and vice versa, with fine-tuning of pH near the IEP. The transfer of polyampholyte dendrimer from anionic to cationic gel in dependence of pH is schematically illustrated in Scheme 11. 

At pH 4.0–4.5 sorption of polyampholyte dendrimer by anionic gel AMPSNa leads to shrinking of the gel layer and concavation of gel sample. At pH 6.5–7.0 the polyampholyte dendrimer is desorbed from AMPSNa and absorbed by PDMDAAC. The formation of Inter-PEC between polyampholyte dendrimer and PDMDAAC gel results in gel contraction and concavation of sample. 

**Figure 17:**
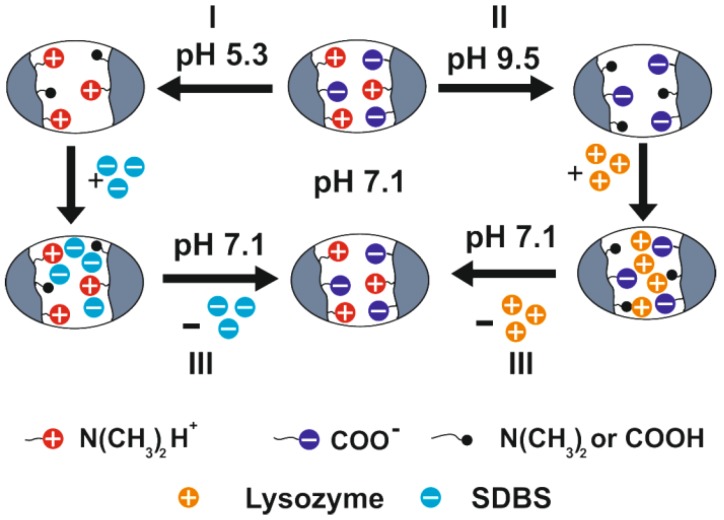
Schematic representation of adsorption of sodium dodecylbenzene sulfonate (SDBS) at pH 5.3 (route 1), lysozyme at pH 9.5 (route 2) and their release at pH 7.1, eg. at the IEP of amphoteric cryogel (route 3). (Reprinted from [155]).

The feasibility of the “isoelectric effect” was tested for crosslinked amphoteric macroporous cryogels consisting of *N*,*N*-dimethylaminoethylmethacrylate and methacrylic acid (DMAEM-*co*-MAA) with respect to surfactant—sodium dodecylbenzene sulfonate (SDBS), dyes methylene blue (MB) and methyl orange (MO), and protein lysozyme [155]. It was found that the release efficiency of dye, surfactant and protein at the IEP from amphoteric cryogel (pH_IEP_ = 7.1) can reach up to 98%. Figure 17 shows release mechanism of low- and high-molecular compounds at the IEP of amphoteric cryogel.

At the IEP of amphoteric cryogel the cooperativity of intrachain interactions between the acidic and basic groups within a cryogel chain exceeds those of interchain interactions between cryogel and protein (surfactant and dye molecules). As a result, at the IEP of amphoteric cryogel (pH_IEP_ = 7.1) the lysozyme, MB, MO and SDBS are released from the cryogel matrix to the outer solution.

An alternative way of protein separation is molecularly imprinted polyampholyte (MIP) hydrogels and cryogels that are synthesized by crosslinking acid-base monomers in the presence of template proteins forming polyampholyte–protein complexes within the network [156,157,158]. After removing of the template a cavity with a complementary shape and selective recognition properties to template molecules is maintained [159]. The stimuli sensitive ability of MIP networks to switch on/off the affinity of the networks for the imprinted molecules by fine-tuning of temperature, pH, and ionic strength is promising for biomedical applications [160,161].

## 6. Amphoteric Behavior of Interpolyelectrolyte Complexes

Polyelectrolyte complexes (PEC) are the products of interaction of weak or strong polyelectrolytes in various combinations [102,103,104,105,106,107,162]. The formation of PEC has no analogues in chemistry of low-molecular-weight compounds because the stabilization of the final products is mainly due to the entropy factor. From the thermodynamic point of view, the complementarity of macromolecules bearing cationic and anionic groups is the driving force of PEC formation that is also responsible for self-organization of biopolymers. Depending on the charge density of interacting polyelectrolytes, concentration, stoichiometry, pH, ionic strength, organic solvent additive PEC particles can be maintained as clear solution, opaque solution, precipitation and gel state. The preparation procedure of PEC films, fibers or coatings includes the dissolution of PEC in ternary system consisting of water, salt and organic solvent. A thin interfacial PEC film can rapidly be formed when two high concentrated polyelectrolytes are mixed [102]. PEC membranes can also be designed on the boundary of water–organic solvents [163,164,165,166,167,168,169]. In recent years the layer-by-layer deposition technique received a lot of interest [170,171,172,173]. The existence of stoichiometric (SPEC) and non-stoichiometric (NPEC) polyelectrolyte complexes is commonly recognized today [162]. As distinct from SPEC, where the molar ratio of oppositely charged polyelectrolytes involved into complexation reaction is 1:1, NPEC are the products of cooperative interpolyelectrolyte reactions between oppositely charged polyelectrolytes with different polymerization degrees or chain lengths taken in non-equivalent ratios. 

**Figure 18:**
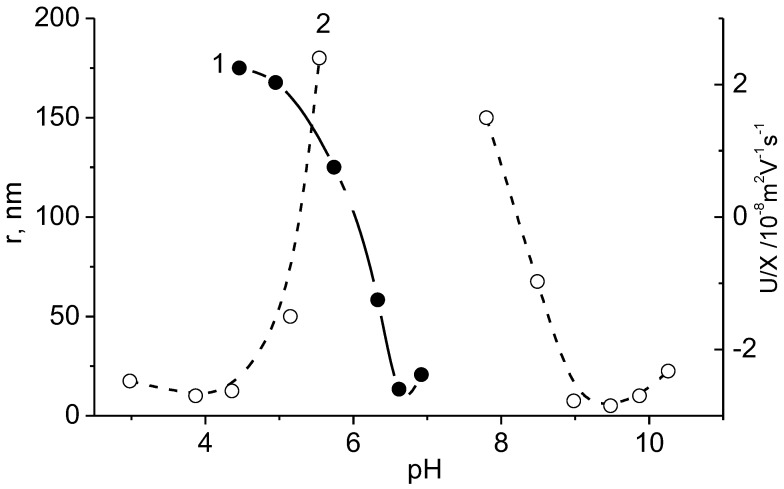
Radius and electrophoretic mobilities of the block polyampholyte PDMAEM-*b*-PMANa (1) and polyelectrolyte complexes of PDMAEM-PMANa (2) as a function of pH at µ = 10^−3^ mol·L^−1^. (Reprinted from Ref. [86]).

Block polyampholytes with the equal number of oppositely charged polyelectrolyte blocks are very close to SPEC, while block polyampholytes with the excess of positively or negatively charged blocks are analogous to NPEC [17,18]. However the main difference between block polyampholytes and PECs is that the interactions between two blocks result in a loss of conformational and translational entropy. The electrophoretic mobilities and radius of the particles of block polyampholyte PDMAEM-*b*-PMANa (the molar ratio of base/acid segment is 1/1.8) and the mixture of PDMAEM and sodium salt of PMAA with the same molar ratio of polybase:polyacid = 1:1.8 are compared in Figure 18 [86].

The IEPs of both systems are 6.0. The instability regions for the mixture of homopolyelectrolytes and the blockpolyampholytes are also similar and displaced near the IEP. However, for the mixture of homopolyelectrolytes the strong complexation starts at 4.5 < pH < 9.0, and for block polyampholyte at 5.5 < pH < 8.0. This fact is probably explained by additional hydrophobic stabilization of interpolyelectrolyte complex particles. The stable homopolymer complexes at pH below 4.5 and above pH 9.0 have smaller size than the copolymer does even if the homopolymer molecules have longer length. 

**Figure 19:**
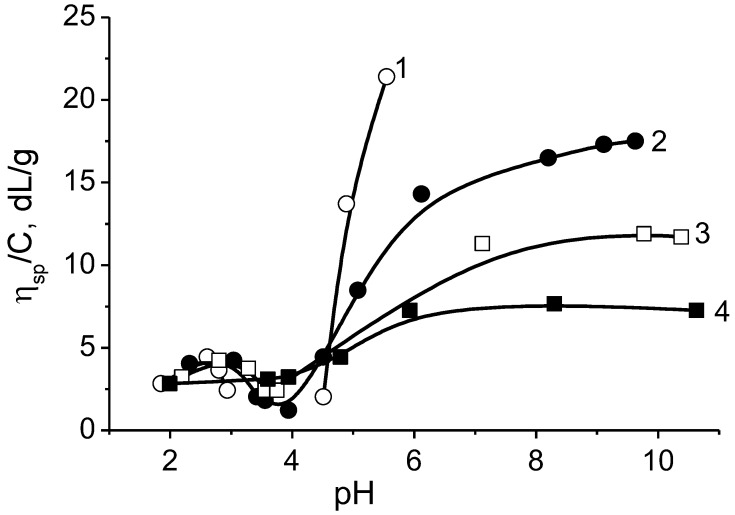
The relationship between the reduced viscosity of the system [poly(sodium acrylate*-co*-acrylamide)]/[poly-2-(*N*,*N*-diethyl-*N*-methylamino)ethyl acrylate] and pH at µ = 0.05 (curve 1), 0.075 (curve 2), 0.25 (curve 3), and 0.5 (curve 4). (Reprinted from [174]).

Isotachophoretic and viscometric results obtained for SPEC consisting of sodium salt of PAA (NaPAA) and poly[2-(*N*,*N*-diethyl-*N*-methylamino)ethyl acrylate] show positive and negative charges at pH = 4.6 and 8.3, respectively, and neutrality at pH = 7.0 [167,168,174,175]. Whereas for NPEC consisting of poly(sodium acrylate-*co*-acrylamide) and poly[2-(*N*,*N*-diethyl-*N*-methylamino)ethyl acrylate] the isoelectric pH corresponds to 4.0. The value of the reduced viscosity at the IEP increases with the growth of the ionic strength as in the case of polyampholytes (Figure 19). 

**Figure 20:**
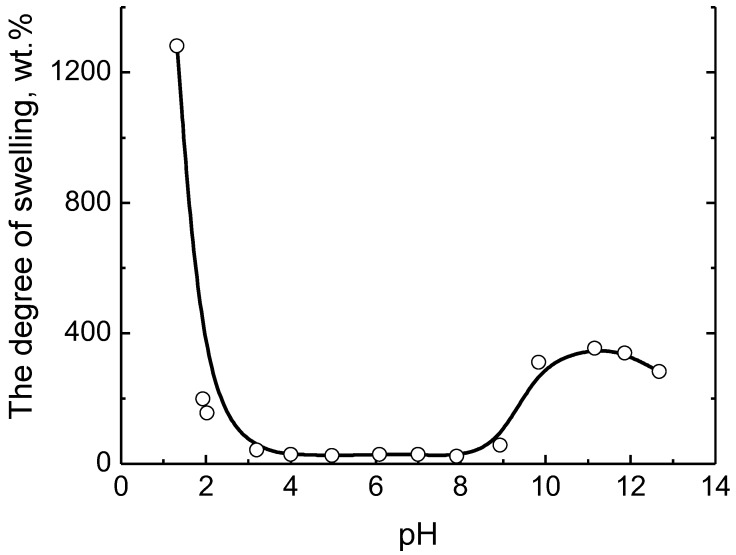
The swelling as a function of pH for semi-IPPN composed of crosslinked chitosan and linear PAA. (Reprinted from [180]).

The conformational behavior of equimolar polyampholytes on the basis of vinyl- and styrenesulfonic acids and aminoalkylmethacrylates [176] repeats the properties of semi-interpenetrating polyelectrolyte networks (semi-IPPN) composed of crosslinked polybase and linear polyacid [177,178,179]. The invariability of the reduced viscosity at 2.8 < pH < 6.0 is an indication of the constant hydrodynamic volume of polyampholyte particles at the IEP [176]. The semi-IPPN [174] also collapses in the range of 3 < pH < 8 due to formation of interpolyelectrolyte complexes between oppositely charged groups. The swelling of semi-IPPN [179] at the IEP with increasing of the ionic strength can be accounted for the screening of ionic network by small ions. PEC membranes derived from the polyelectrolyte complexes [166,167,168,169,180,181] also show amphoteric character [182]. A minimum swelling degree of the membranes is ensured by Coulombic attractions between long sequences of acidic and basic groups of polyelectrolytes (Figure 20). The isoelectric point of PEC solution and membrane where the macromolecule is electroneutral is observed at pH ≅ 6.5. 

Shashua [183] observed the oscillation of the electric potential across an ultrathin PEC membrane designed from *co*(acrylic acid-acrylamide) and PDMAEA in the presence of 0.15M NaCl. Shashua’s membrane consists of a cation selective polyacidic zone (a), an anion-selective polybasic zone (b) and a neutral polyampholytic zone (c). Under an externally imposed potential across the membrane, the cations will migrate from left to right while the anions will act oppositely. The salt accumulation takes place in the ampholytic region. Such salt accumulation invokes two effects. The generated osmotic pressure in the ampholytic zone (c) will cause water flow and induce an increase of hydrostatic pressure. Simultaneously, the accumulated salt will cause the contraction of polyanionic (a) and polycationic (b) zones. If the hydrostatic pressure generated from the polyelectrolyte configurational change overcomes the osmotic pressure, the solvent flow will be reversed. The process can be regenerated with the addition of salt which subsequently forces solvent from the zone c, thereby driving the concentration yet higher. In the regenerative step, salt will move out of the ampholytic region because of its high concentration gradient, and will continue its flow out until the membrane has reached the point of maximum contraction. Thus the process moves into the stage of diminishing salt concentration, following the relaxation of the PEC membrane matrix, and eventual return of the membrane to its initial state. The analytical solution of this phenomenon has been presented.

## 7. Conclusions

Intra- and interpolyelectrolyte complexes of polyampholytes represent an unusual category of the polyelectrolyte complexes (PEC) that are the products of interaction of oppositely charged polyelectrolytes. The formation of polyelectrolyte-polyampholyte complexes (PPC) with participation of random, regular, block, dendritic polyampholytes has no analogues in chemistry of low-molecular-weight compounds because the stabilization of final products is mainly due to electrostatic attraction between the opposite charges and entropy factor arising from the release of counterions. As evidenced in recent experiments [184,185], the driving entropy forces also originate from the rearrangement of water molecules around the polymer chains during the complexation. The complementarity of interacting macromolecules bearing charged groups is the driving force of PPC formation that is also responsible for the self-organization of biopolymers. In a dependence with charge density of interacting components, concentration, stoichiometry, pH, ionic strength, organic solvent additives, the PPC can be formed as clear solution, opaque solution, precipitation and gel state. As distinct from homopolyelectrolytes, polyampholytes can interact with both anionic and cationic macromolecules. This is especially specific for block polyampholytes. In turn the complexation of the equal number of oppositely charged polyelectrolyte blocks are very close to the formation of stoichiometric PEC, while block polyampholytes with the excess of positively or negatively charged blocks are able to form non-stoichiometric PEC. However the main difference between blockpolyampholytes and PECs is a loss of conformational and translational entropy caused by the interaction between two blocks. The stability order of PPC is theoretically predicted and experimentally confirmed as shown in the following order: block polyampholyte–polyelectrolyte > statistical polyampholyte–polyelectrolyte > alternating polyampholyte–polyelectrolyte. In most cases the suggested theoretical models describe the solution behavior and complexation properties of polyampholytes reasonably well. Several fundamental properties of polyampholytes were discovered in the last century. One of them is the so-called “antipolyelectrolyte” effect predicted by Ehrlich and Doty [186], theoretically concluded by Higgs and Joanny [42] and confirmed experimentally by many research groups [187,188,189,190]. The “antipolyelectrolyte” effect is related to the hydrodynamic size (or unfolding) of amphoteric macromolecules at the isoelectric point (IEP) upon addition of low-molecular-weight salts. Afterwards, this statement was confirmed for statistical-, alternating- and block-polyampholytes of various natures and structures as well as for polyampholyte hydrogels. Another fundamental discovery is the so called “isoelectric effect” described in subsection 5. Briefly, any substances bound to polyampholytes may be discharged at the IEP via governing forces among inter- and intramolecular paths. Furthermore, as the intrachain interaction of acidic and basic groups within a single macromolecule is superior to the interchain interaction, releasing the loaded substance from the polyampholytes carrier can be fulfilled. The realization of the “isoelectric effect” for a series of water-soluble polyampholytes, amphoteric hydrogels and cryogels was demonstrated with respect to surfactants, dye molecules, polyelectrolytes and proteins. The third finding is the structural and behavioral similarity of stoichiometric and non-stoichiometric polyelectrolyte complexes as well as interpenetrating polyelectrolyte networks to polyampholytes that is formulated as follows: (1) stoichiometric and nonstoichiometric PEC can serve as model of balanced or neutral (the same number of negative and positive charges) and unbalanced or non-neutral (an excess of positive or negative charges) polyampholytes; (2) stoichiometric and nonstoichiometric PEC have a compact structure and minimum solubility at the isoelectric pH; (3) near the IEP both PECs and polyampholytes are able to swell at high salinity media; (4) the oscillation mechanism of PEC membranes and polyampholyte gels has the same nature and explanation; (5) the “isoelectric” effect found at the IEP of polyampholytes is also observed for PEC. An open problem in the behavior of polyampholytes is the globular structure of macromolecules that can be considered as dense three-dimensional nucleations consisting of a “core” from which the monomers “force out” the solvent and surrounded by a hydrophilic “edge”. The possibility of formation of a highly ordered (crystaline) structure at the IEP of water-soluble polyampholytes is also a challenging task. Here, theoretical, computational and simulation approaches for better understanding of intra- and interpolyelectrolyte complexation of polyampholytes are required.

## Figures and Tables

**Table 1:** Examples of various polyampholytes.

Chemical Structure of Monomer Units	Name Acronym	Type of Polyampholyte	Refs
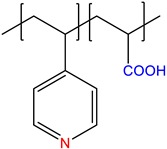	4-vinylpyridine-*co*-acrylic acid, P(4VPy-*co*-AA)	Annealed	[12]
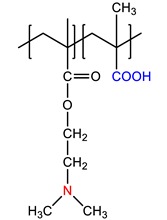	*N*,*N*-dimethylaminoethylmethacrylate- *co*-methacrylic acid, P(DMAEM-*co*-MAA)	Annealed	[13]
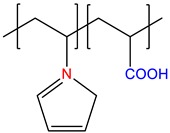	*N*-vinylimidazole-*co*-acryic acid, P(VI-*co*-AA)	Annealed	[14]
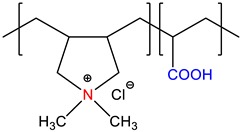	*N*,*N*’-dimethyl-*N*,*N*’-diallylammonium chloride-*co*-acrylic acid, P(DMDAAC-*co*-AA)	Self-quenched *	[15]
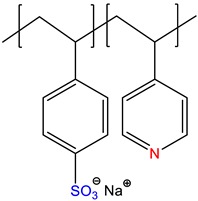	sodium styrene sulfonate-*co*-4-vinylpyridine, P(NaSS-*co*-4VPy)	Self-quenched *	[16]
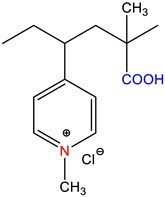	poly(1-methyl-4-vinylpyridinium chloride)-*block*-poly(methacrylic acid), P1M4VPCl-*b*-PMAA	Self-quenched *	[17,18]
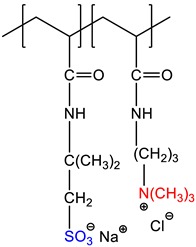	2-acrylamido-2-methyl-1-propanesulfonic acid sodium salt-*co*-(3-acrylamidopropyl)trimethylammonium chloride, P(AMPSNa-*co*-APTAC)	Quenched	[19]
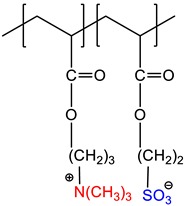	2-methacryloyloxyethyltrimethylammonium-*co*-2-methacryloyloxyethanesulfonate, P(METMA-*co*-MES)	Quenched or zwitterionic	[20]
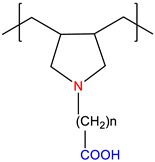	Poly(carbobetaine), PCB	Annealed	[7]
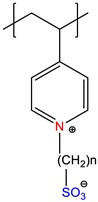	Poly(sulfobetaine), PSB	Quenched or zwitterionic	[7]
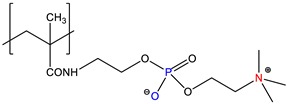	Poly(phosphobetaine), PPB	Quenched or zwitterionic	[7]

* The term “self-quenched” for polyampholytes represents polyampholytes which possess permanently ionized charged groups (anionic or cationic) in the repeating units.

**Table 2:** Composition of polyelectrolyte-polyampholyte complexes (PPC) formed between APA and PAA and NaPSS.

Polyampholytes	Polyelectrolytes (PE)	PPC, *n* = [APA]/[PE]
APA-1	PAA	1:1
	NaPSS	3:1
APA-2	PAA	1:4
	NaPSS	5:2
APA-3	PAA	1:2
	NaPSS	5:1
APA-4	PAA	1:2
